# STING in tumors: a focus on non-innate immune pathways

**DOI:** 10.3389/fcell.2023.1278461

**Published:** 2023-10-25

**Authors:** Jiaying Yang, Mei Yang, Yingtong Wang, Jicheng Sun, Yiran Liu, Ling Zhang, Baofeng Guo

**Affiliations:** ^1^ Department of Plastic Surgery, China-Japan Union Hospital, Jilin University, Changchun, China; ^2^ Key Laboratory of Pathobiology, Ministry of Education, and Department of Biomedical Science, College of Basic Medical Sciences, Jilin University, Changchun, China

**Keywords:** mtDNA, cGAS-STING, PD-L1, TME, extracellular vesicles

## Abstract

Cyclic GMP-AMP synthase (cGAS) and downstream stimulator of interferon genes (STING) are involved in mediating innate immunity by promoting the release of interferon and other inflammatory factors. Mitochondrial DNA (mtDNA) with a double-stranded structure has greater efficiency and sensitivity in being detected by DNA sensors and thus has an important role in the activation of the cGAS-STING pathway. Many previous findings suggest that the cGAS-STING pathway-mediated innate immune regulation is the most important aspect affecting tumor survival, not only in its anti-tumor role but also in shaping the immunosuppressive tumor microenvironment (TME) through a variety of pathways. However, recent studies have shown that STING regulation of non-immune pathways is equally profound and also involved in tumor cell progression. In this paper, we will focus on the non-innate immune system pathways, in which the cGAS-STING pathway also plays an important role in cancer.

## 1 Introduction

cGAS is highly conserved and is the double-stranded DNA (dsDNA) sensor, which is responsible for monitoring the changes in cytoplasmic DNA content in most mammalian cells (Sun et al., 2013; Ablasser and Chen, 2019). In contrast to Toll-like receptor 9 and NLRP3, whose expression appears to be restricted to immune cells, cGAS and the downstream STING signaling are widely expressed in most cell types (Chen et al., 2016a; Riley and Tait, 2020). It has been shown that cytoplasmic leakage of mtDNA is the major source of cytoplasmic dsDNA in ATM-deficient tumor cells (Hu et al., 2021a), suggesting that mtDNA may play an important role in the innate immunity of tumor cells. The mtDNA is located near the five complexes that transfer electrons across the inner mitochondrial membrane (IMM) to ensure efficient synthesis of the proteins encoded by the mitochondrial genes, but the electron transport chain as a major source of mitochondrial reactive oxygen species (ROS) will result in mtDNA being more susceptible to oxidative damage and leakage into the cytoplasm. The cyclic structure of mtDNA and the presence of unmethylated CpG motifs allow cGAS to recognize mtDNA leakage into the cytoplasm as a “foreign” molecule that triggers an inflammatory response (West and Shadel, 2017; Kausar et al., 2020; De Gaetano et al., 2021), which is then involved in tumorigenesis through subsequent STING signaling. By analyzing The Cancer Genome Atlas of 18 malignancies, several scientists found that the expression of four key molecules in the cGAS-STING pathway, MB21D1 which encodes cGAS, TMEM173 which encodes STING, as well as TANK-binding kinase 1 (TBK1) and interferon-regulating factor 3 (IRF3), are significantly upregulated in almost all detected cancer types compared to normal control tissues, suggesting that cGAS-STING and the signaling molecules may be activated in all cancer processes (An et al., 2019).

It is now widely accepted that the cGAS-STING signaling cascade may rely primarily on synthetic interferons (IFNs) to exert anti-tumor effects, but recent studies have found that it also has significant regulatory effects on non-immune pathways and influences tumor progression. At the same time, it has been found that there is significant heterogeneity in the outcomes exhibited by tumor cells upon STING activation, and the mechanism of this double-edged role in tumor progression deserves to be explored. Therefore, in this review, we summarize the main mechanisms of mtDNA escaping from mitochondria and the recent advances in the impact on tumor cells after the occurrence of horizontal metastasis, with a focus on the role of the subsequent non-innate immunoregulatory pathways of the cGAS-STING pathway in tumor progression and the potential factors that influence STING bi-directionality.

## 2 Overview of cGAS-STING

cGAS is a member of the nucleotidyltransferase family and is normally present in the cytoplasm as an inactive protein, and current evidence suggests that cGAS also has a diverse cellular distribution (Kuchta et al., 2009; Sun et al., 2013; Wu et al., 2013; Hopfner and Hornung, 2020). Jiang et al. showed that cGAS is also constitutively present in the nucleus. When it is activated, it accelerates genomic instability, the formation of micronuclei (MNi), and cell death under stress conditions by inhibiting homologous recombination pathways (Jiang et al., 2019). This dual function of cGAS as an innate immune sensor in the cytoplasm and a negative regulator of DNA repair in the nucleus highlights the importance of cGAS in cells. Briefly, After the recognition and binding of free cytoplasmic DNA to form the complexes in the cytoplasm, there has been a conformational change in the active site of cGAS, which causes cGAS activation. Then the cyclic guanosine monophosphate-adenosine monophosphate (cGAMP) is synthesized by cGAS with intracytoplasmic ATP and GTP as raw materials. As the secondary messenger, cGAMP or cyclic dinucleotides (CDNs) bind to the adapter protein STING anchored to the endoplasmic reticulum (ER) (Ablasser et al., 2013; Gao et al., 2013a; Diner et al., 2013; Sun et al., 2013; Zhang et al., 2013). Upon binding to cGAMP, STING undergoes extensive conformational rearrangements (Huang et al., 2012; Shang et al., 2012; Shu et al., 2012; Gao et al., 2013b; Ergun et al., 2019; Shang et al., 2019). After conformational rearrangement, STING is translocated from the ER to the Golgi through the ER-Golgi intermediate compartment (Dobbs et al., 2015). Upon arrival at the Golgi compartment, STING is palmitoylated at two cysteine residues (Cys88 and Cys91) and recruits TBK1 (Mukai et al., 2016). TBK1 then phosphorylates itself. STING and IRF3 dimerize and enter the nucleus, triggering type I interferon production (Hopfner and Hornung, 2020). Phosphorylated TBK1 and its cognate IκB kinase (IKK) also mediate activation of the IKK complex, which in turn activates the atypical NF-κB pathway to induce the production of other inflammatory cytokines (Smale, 2010). For detailed mechanisms, see the excellent review by Hopfner and Hornung (2020).

## 3 Regulation and modification of cGAS and STING expression

cGAS is constitutively expressed in most cell types. Thus, unlike other pattern recognition receptor systems, the levels of cGAS and its ability to be activated are not regulated at the transcriptional level. Conversely, many post-translational mechanisms have been shown to play an important role in regulating the activity of this receptor toward its ligand, enzymatic activity, or half-life (Hopfner and Hornung, 2020). cGAS and STING undergo several types of post-translational modifications, including protein hydrolysis (Wang et al., 2017), acetylation (Dai et al., 2019), glutamylation (Xia et al., 2016a), ubiquitination (Zhang et al., 2012), sumoylation (Hu et al., 2016), and phosphorylation (Tanaka and Chen, 2012). These post-translational modifications affect cGAS and STING either by direct modification or by cleavage of active site residues. In particular, STING signaling is defective in a variety of cancers (e.g., colon cancer and melanoma), and its behavior may allow damaged cells to escape immune surveillance (Xia et al., 2016b; Xia et al., 2016c). Konno et al. found that cGAS or STING genes were mutated in a variety of human tumors by searching the cBioPortal database. Among them, cGAS missense mutants R376Q and E383K lost the ability to produce cGAMP upon binding to dsDNA, while STING mutants (R169W and P203S) were unable to promote the production of oncogene-induced pro-inflammatory cytokines. Hypermethylation of cGAS or STING promoter regions is present in several types of tumor samples, resulting in cGAS or STING repression (Konno et al., 2018). It has been shown that this methylation process is reversible, and in STING-deficient melanoma cell lines, demethylation-mediated restoration of STING signaling can increase the antigenicity of MHC-like molecules by upregulating them, thereby enhancing their recognition and killing by cytotoxic T cells (Falahat et al., 2021).

## 4 mtDNA is a potent inducer of the cGAS-STING pathway

One of the characteristics of tumor cells is the massive replication of chromosomes in the nucleus, which can lead to the leakage of genomic DNA into the cytoplasm (Chin et al., 2020). When the mitotic process of cells is damaged endogenously or exogenously, some chromatin segments may be missegregated into the cytoplasm, either passively or actively, forming one or more spatially separated MNi (Guo et al., 2019). Gupta et al. found Cisplatin-induced massive MNi production in cutaneous squamous carcinoma (Gupta et al., 2011). Using high-resolution live-cell imaging, Hatch et al. found that spontaneous rupture of the micronuclear envelope can occur in the majority of MNi (Hatch et al., 2013), and that ruptured MNi are an important source of cytoplasmic self-DNA (mainly dsDNA) (Harding et al., 2017). Approximately 55% of metastatic tumor-derived cells were reported to be cGAS positive (Bakhoum et al., 2018). Similarly, cytoplasmic translocation of mtDNA has been detected in many disease states and has been associated with cancer progression (Liu et al., 2019b; Piantadosi, 2020). In particular, when tumor cells are often subjected to multiple physicochemical insults during treatment, their mitochondrial damage is further aggravated and more mtDNA is released into the cytoplasm (Cheng et al., 2020; Zhu et al., 2022). Numerous studies have shown that mtDNA is a potent inducer of cGAS-STING signaling (Wu et al., 2021b). Interestingly, Tigano et al. used the inducible restriction endonuclease AsiSi to selectively induce DNA double-strand breaks (DSBs) in the nucleus and ionization radiation (IR) to induce DSBs in both mtDNA and nuclear DNA. The results showed that although AsiSi and IR caused the cells to produce similar levels of cGAS-positive MNi (∼15%), the induction of nuclear DNA and mtDNA co-damage showed stronger interferon-stimulated gene activation and significantly increased transcript levels than the induction of nuclear DNA damage alone. This suggests that damaged mtDNA synergizes with MNi to produce a stronger type I IFN response (Tigano et al., 2021). The reason for the strong activation of cGAS by mtDNA may be related to the fact that mtDNA is more susceptible to damage due to its high copy number and inefficient repair system (Wu et al., 2021a). In addition, the structural features of mtDNA, i.e., shorter circular DNA, and no histone hindrance, seem to be the critical factors (Andreeva et al., 2017). However, it has also been shown that cytoplasmic chromatin is equally competent for cGAS-STING activation (Dou et al., 2017). Structurally, the N-terminus of cGAS is required for nuclear chromatin sensing, not mtDNA (Li et al., 2021c). Compared with naked dsDNA, reconstituted nucleosomes *in vitro* have higher affinities for cGAS, but lower capacities to activate it (Wang et al., 2020a). The reason for this may be that nucleosomes interfere to some extent with the formation of this DNA structure, and thus some studies have used nucleosomes as markers to distinguish their DNA from pathogenic DNA (Zierhut et al., 2014; Zierhut and Funabiki, 2015). Given that mtDNA is an important component of dsDNA and plays an important role in cGAS-STING activation, the next section will explore the main mechanisms of mtDNA leakage into the cytosol in the context of the current findings.

## 5 The main mechanism of mtDNA leakage

Mitochondrial outer membrane permeability (MOMP), initiated by endogenous or mitochondrial apoptotic processes, is one of the major forms of mtDNA release. Previous studies have long shown that free Bax and Bak oligomerize in the outer mitochondrial membrane (OMM) during apoptosis to form a proteic pore, which can lead to the release of mitochondrial contents such as cyt C, mtDNA, *etc.*, into the cytosol (Chipuk et al., 2006). However, since the IMM is thought to remain intact during apoptosis (Bernardi et al., 2015), it is particularly mysterious how mtDNA escapes from the mitochondria during this process. McArthur et al. examined mitochondria from mouse embryonic fibroblasts using live-cell lattice light-sheet microscopy and found that BAX/BAK activation and subsequent cyt C release are followed by mitochondrial lattice rupture and the appearance of large BAX/BAK pores on the OMM. These large pores allow IMM-carrying mitochondrial matrix components, including the mitochondrial genome, to protrude into the cytosol, ultimately leading to the release of contents such as mtDNA (McArthur et al., 2018). The study by Riley et al. also confirmed the above and indicated that after the occurrence of BAX-dependent MOMP, mitochondrial inner membrane permeability (MIMP) may also follow (Riley et al., 2018). It is noteworthy that tBID, a member of the BH3-only family, can mediate MOMP in a BAX/BAK-independent manner. The finding defines tBID as an effector of mitochondrial permeability in apoptosis (Flores-Romero et al., 2022) and may influence the process of mtDNA release through MOMP.

Another important pathway for mtDNA escape from mitochondria is the opening of the mitochondrial permeability transition pore (mPTP). The opening of mPTP leads to increased IMM permeability and thus to changes in mitochondrial permeability (Bernardi and Di Lisa, 2015; Bernardi, 2018). It has been shown that mtDNA fragments are released from the brains of irradiated mice by briefly opening the mPTP (Patrushev et al., 2006). Under oxidative stress conditions, DNA from liver mitochondria can also be released by nonspecific mPTP. In the presence of Ca^2+^, the addition of Fe^2+^ and H_2_O_2_ induces the opening of mPTP, and mtDNA is observed to be hydrolyzed after oxidative stress, with partial release of mDNA fragments into the cytoplasm (Garcia et al., 2005). Different studies have shown differences in the amount of mtDNA fragments released. Garcia et al. found that the DNA content in the mitochondrial matrix decreased to 42% ± 6% and the number of MTCO1, MTND3, and MTCYB genes in the mitochondria decreased by approximately 46%, 22%, and 54%, respectively, and mtDNA release could be inhibited in the presence of cyclosporin A, an mPTP inhibitor (Garcia and Chavez, 2007). In summary, mtDNA can be released by mPTP, and incomplete mtDNA fragments are released, which is consistent with the fact that mPTP only allows the transport of molecules<1.5 kDa (Halestrap et al., 2002). Recent studies have revealed the mechanism by which this process occurs. When mitochondrial stress occurs, ROS induces the production of oxidized mtDNA (Ox-mtDNA), which can be repaired by the DNA glycosylase OGG1, and when the repair mechanism is limited, Ox-mtDNA is cleaved within the mitochondria by the nucleic acid endonuclease FEN1 into 500–650 bp fragments, which leave the mPTP- and voltage-dependent anion channel-(VDAC) dependent channels through the mitochondria, thus initiating the activation of inflammation (Xian et al., 2022; Cabral et al., 2023). During this time, VDAC plays a role in helping mtDNA open the door to the cytoplasm. VDAC, also known as mitochondrial porin, is the major protein involved in transporting OMM (Shoshan-Barmatz et al., 2006). One of the VDAC-1 isoforms can form oligomers on the OMM (Keinan et al., 2010). Kim et al. found in the mice model of systemic lupus erythematosus (SLE) that when mitochondria are stressed in multiple ways, the fragmented mtDNA binds to the VDAC on the OMM, which causes multiple VDAC monomers to cluster together and form a mesopore in their middle, allowing mtDNA to be released into the cytoplasm through this mesopore by direct interaction between the three positively charged residues at the N-terminal end of the VDAC and mtDNA (Kim et al., 2019). Researchers applied the oligomerization inhibitor VBIT-4 to block only one of the channel forms, VDAC1, to reduce mtDNA release in the mice model of SLE, demonstrating the potential role of VDAC in mtDNA release (Kim et al., 2019). In conclusion, when oxidative stress damages mitochondria, it triggers the opening of mPTP on IMM while oxidizing mtDNA. Ox-mtDNA undergoes shearing by nucleic acid endonucleases and then enters cytoplasmic lysis through mPTP and VDAC on OMM in sequence, which in turn triggers subsequent inflammatory responses. It is worth mentioning that the structural opening of the pores may also lead to mitochondrial swelling, resulting in IMM disruption and thus cytosolic release of mtDNA (Riley and Tait, 2020). Whether this effect of mPTP on IMM is related to MIMP needs to be further explored. Li et al. also showed that the inherent tripartite motif 21 of tumor cells promotes VDAC2 degradation through K48-linked ubiquitination, which inhibits the pore-forming capacity of VDAC2 oligomers and reduces mtDNA leakage, which in turn inhibits the type I interferon response following exposure to IR (Li et al., 2023). Notably, the structural opening of the pore can also lead to mitochondrial swelling and IMM disruption, which promotes the release of mtDNA into the cytosol (Riley and Tait, 2020). Whether this effect of mPTP on IMM is related to MIMP deserves further investigation. It has been shown that chitosan, a vaccine adjuvant, activates the cGAS-STING pathway and induces the type I IFN response via mPTP-dependent release of mtDNA (Carroll et al., 2016). These results suggest that mPTP may play a more important role in activating the cGAS-STING pathway, and although the structural integrity of mtDNA is difficult to maintain, this fragmented DNA does not affect the recognition of cGAS.

Gasdermins (GSDMs) play a key role in pyroptosis. Its N-terminal structural domain can form transmembrane pores in the plasma membrane, leading to changes in membrane permeability (Chen et al., 2016b; Ding et al., 2016; Liu et al., 2016). Besides the plasma membrane, there is evidence that the GSDM family can form pores in the mitochondrial membrane. Weindel et al. also found that in macrophages, elevated mitochondrial ROS (mtROS) direct the binding of GSDMD to the mitochondrial membrane, followed by the formation of mitochondrial GSDMD pores and then the release of mtROS (Weindel et al., 2022). Huang et al. showed that lipopolysaccharide (LPS) activates GSDMD and forms a mitochondrial pore that induces the release of mtDNA into the cytoplasm of endothelial cells to be recognized by cGAS (Huang et al., 2020). These findings suggest that GSDM may be involved in the release of mtDNA as a novel pathway.

In addition, changes in mitochondrial dynamics contribute to the release of mtDNA from the mitochondria. Mitochondrial transcription factor A (TFAM) plays an important role in mitochondrial quality control, and imbalances in TFAM and/or mtDNA homeostasis are frequently observed in tumor cells (Wallace, 2012). West et al. showed that TFAM-deficient fibroblasts display elongated and highly fused mitochondria and increased cytosolic mtDNA (West et al., 2015). Similarly, inhibition of ataxia-mutated protein (ATM) leads to downregulation of TFAM, allowing mtDNA to escape to the cytoplasm (Hu et al., 2021b). Mitochondrial division is important to ensure proper nucleoid distribution and removal of damaged mtDNA (Meyer et al., 2017). It has been shown that activation of STING signaling in Kupffer cells after LPS stimulation may be associated with enhanced mitochondrial division and increased mtDNA release caused by upregulation of dynamin-related protein 1 (DRP1) (Zhang et al., 2023c). Furthermore, LPS enhanced DRP1-dependent mtROS production, which further enhanced STING signaling by promoting mtDNA leakage into the cytosol (Li et al., 2022; Zhang et al., 2023c). Similarly, a recent study showed that the knockdown of GTPase—MxB, a member of the membrane-deforming dynamin family located in the IMM, resulted in mitochondrial fragmentation, IMM breakage, and increased cytoplasmic mtDNA levels (Cao et al., 2020). This suggests that homeostatic imbalance is likely another factor in the release of mtDNA into the cytoplasm.

## 6 Presence of intercellular transfer of mtDNA, cGAS, and STING

**FIGURE 1:**
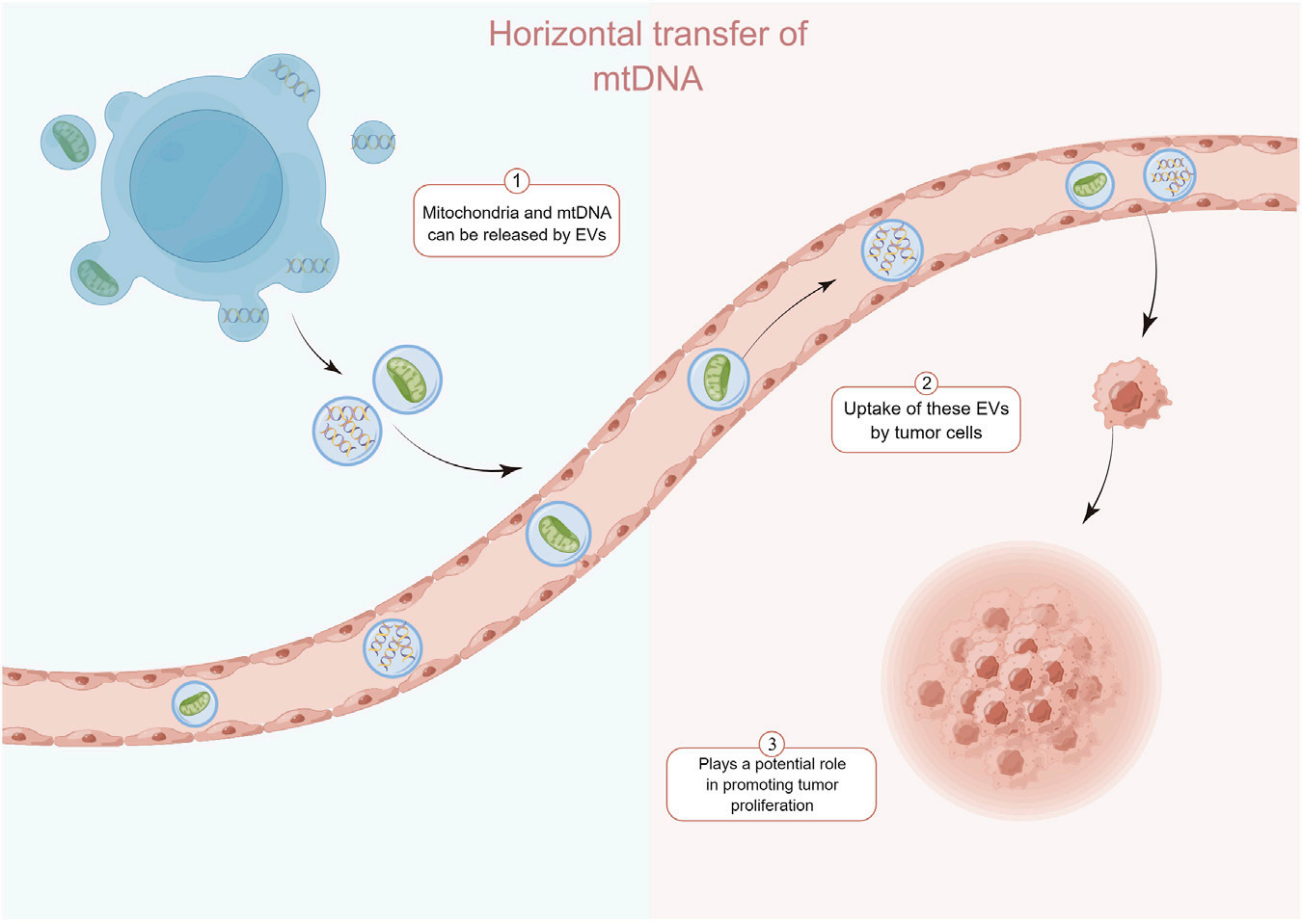
Normal cell-derived mtDNA and mitochondria can be transferred to tumor cells via the extracellular vesicle pathway and enhance the recovery of their tumorigenic potential. By Figdraw.

In recent years, horizontal transfer of mitochondria and mtDNA between tumor cells and surrounding non-tumor cells has been reported. Tumor cells acquire functional mitochondria and mtDNA from healthy host cells to repair or even enhance impaired mitochondrial function, thereby restoring tumorigenic potential. Tumor cells show a significant delay in tumor growth in the absence of mtDNA (Rebbeck et al., 2011). Salaud et al. found that mitochondria can be transferred from tumor-activated stromal cells present in the glioblastoma (GBM) microenvironment to GBM, and this transfer increases the proliferation of GBM and its resistance to radiotherapy and chemotherapy (Salaud et al., 2020). This transfer of intact mitochondria from host cells may be an important way for tumor cells to acquire mtDNA (Tan et al., 2015; Dong et al., 2017) (Figure 1). Interestingly, Kleih et al. found that cisplatin-sensitive high-grade plasmacytoid ovarian cancer cell lines contained higher mitochondrial content than cisplatin-resistant cells (Kleih et al., 2019). This suggests that mtDNA or mitochondria may play different roles depending on the specific context and stage of tumor progression.

The transfer of mtDNA through “tunneling nanotubes” (TnTs) may be an important way of cross-talk between stromal cells and cancer cells (Pasquier et al., 2013). In addition to the TnTs pathway of cellular level translocation of mtDNA, the extracellular vesicle (EV) pathway is another important way of mtDNA level transfer. Circulating EVs from breast cancer patients identify complete mitochondrial genomes (Sansone et al., 2017). Rabas et al. also found that in breast cancer cells, PINK1 drives the production of mtDNA-loaded EVs and transfers invasive properties to “recipient” tumor cells (Rabas et al., 2021). Recent studies have identified mechanisms that regulate the translocation of mitochondrial components, including mtDNA, into EVs. Mitochondria-derived vesicles can transport components of the mitochondrial interior to nearby organelles. This process is dependent on the proteins optic atrophy 1 and categorical connexin 9 (Todkar et al., 2021). Notably, the presence of mitochondrial proteins in EVs has been demonstrated in the absence of pro-inflammatory stimuli (Hurwitz et al., 2016; Kowal et al., 2016). So, is it possible that mitochondria regulate tumor cells survival by actively releasing mtDNA-containing EVs, or is the acquisition of mtDNA by tumor cells a “voluntary act” that occurs when the tumor cells force the normal cells through certain pathways? Besides mitochondria and mtDNA, activated cGAS and STING can be transferred to recipient cells via EVs. Clancy et al. found that a portion of the dsDNA coupled to activated cGAS was encapsulated in tumor microvesicles (TMVs) and that the TMVs transferred both to the recipient cells and influenced the behavior of the recipient cells (Clancy et al., 2022). In addition, RAB22A mediates the formation of atypical autophagosomes containing STING activated by agonists or radiotherapy, while RAB22A inactivates RAB7 and inhibits the fusion of formed autophagosomes with lysosomes, resulting in the release of endo-vesicular vesicles of autophagosomes with activated STING into the extracellular space (Gao et al., 2022). It is foreseeable that this direct cell-to-cell transfer of activators may have a faster and more direct effect than the transfer of DNA, leading to different consequences.

## 7 STING exerts anti-tumor effects through the innate immune pathway

Given the role of the cGAS-STING pathway in activating immune surveillance, current studies have focused on innate immune function to exert anti-tumor effects (Li et al., 2019; Kwon and Bakhoum, 2020). Due to the inherent genomic instability of cancer cells, they can display constitutive activation of intrinsic immunity and cGAS-STING pathway-mediated IFN signaling, with the production of IFN-a and IFN-b that bind to IFN receptors on themselves, neighboring cells or immune cells, mobilizing immune cells such as dendritic cells (DCs) and TME infiltration of CD8^+^T cells to eliminate the tumor (Diamond et al., 2011; Fuertes et al., 2011; Reislander et al., 2020). Grabosch et al. activated the cGAS-STING pathway in ovarian cancer mice using the DNA damage inducer cisplatin and increased CD8^+^T cells infiltration (Grabosch et al., 2019). Chemosuppression of ATM activated the cGAS-STING signaling pathway by downregulating TFAM to promote mtDNA release, which similarly enhanced T lymphocytes infiltration into TME (Hu et al., 2021b). Harabuchi and others used a combination of cisplatin and cGAMP to increase chemokine expression in tumor tissue, transform tumors from “cold” to “hot” via STING, increase CD8^+^T cells infiltration, and significantly inhibit tumor growth in tumor-bearing mice (Harabuchi et al., 2020). This transformation to “hot” tumors may be associated with the expression of chemokines, such as CXCL9, CXCL10, CCL5, CCL20, *etc.*, which recruit antigen-presenting cells (APCs), T cells, and NK cells to infiltrate the TME and kill tumor cells (Corbera-Bellalta et al., 2016; Hu et al., 2023).

## 8 STING exerts anti-tumor effects through non-innate immune pathways

### 8.1 STING regulated autophagy

Induction of autophagy is one of the important functions of STING and may play a role in the interferon signaling process before STING (Gui et al., 2019; Nassour et al., 2019). Nassour et al. showed that STING-driven macroautophagy, a subtype of autophagy involving autophagic lysosomes, is a critical step in preventing cancer development (Nassour et al., 2019). Liu et al. reported STING-induced atypical autophagy dependent on ATG5 (Liu et al., 2019a). Typical autophagy can also be triggered by the interaction of cGAS and Beclin-1, fluid-phase separation of cGAS-DNA complexes, and STING-triggered ER stress-mTOR signaling (Zhang et al., 2023c). Autophagy-dependent cell death can be triggered when DNA damage-induced STING activation clears cancer cell (Zhang et al., 2021).

### 8.2 STING-regulated cell death pathways

The molecular mechanisms behind STING-associated cell death involve multiple signaling cascades. Ferroptosis is a form of cell death characterized by iron-mediated membrane lipid peroxidation and dysregulation of anti-oxidant levels (Tang et al., 2021). The nucleoside analog zalcitabine induces mitochondrial damage and mtDNA release, activates the cGAS-STING pathway, which in turn induces autophagy-dependent ferroptosis and inhibits pancreatic tumor growth in mice (Li et al., 2021b). STING also promotes ferroptosis in human pancreatic cancer cell lines by increasing mitofusin 1/2-dependent mitochondrial fusion, which induces massive ROS production and ultimately membrane lipid peroxidation (Li et al., 2021a). Zierhut et al. showed that cGAS activation is inhibited by nucleosomes during normal mitosis, but during prolonged mitotic arrest, cGAS promotes a slow accumulation of IRF3 phosphorylation, which in turn may contribute to apoptosis by inhibiting Bcl-xL. This ability to exert inflammatory signaling in transcriptionally competent interphase cells while inducing apoptosis in transcriptionally attenuated mitotic cells contributes to increased sensitivity to paclitaxel, a chemotherapeutic agent that acts by inhibiting cell mitosis, in patients with lung and ovarian cancer (Zierhut et al., 2019). Since certain types of DNA damage can lead to mitotic blockage and mitotic cell death (Garner et al., 2013), cGAS may also affect other types of anti-tumor responses that use DNA damage as a mechanism of action. For example, Banerjee et al. found that STING affects cell death through the DNA damage response (DDR) system independently of the typical IFN pathway. The STING-TBK1 axis stimulates phosphorylation of the kinase ATM, which activates the CHK2-p53-p21 pathway and induces apoptosis by blocking the cellular G1 phase (Banerjee et al., 2021). In addition, a genome-wide CRISPR-Cas9 screen by Hayman et al. revealed that STING acts as a regulator of ROS homeostasis that deletion of STING leads to enhanced cellular ROS metabolism and therapeutic resistance to DNA damaging agents in head and neck squamous cell carcinoma, and that pharmacological activation of STING enhances the anti-tumor effects of IR *in vivo*. A rationale for the therapeutic combination of STING agonists and DNA-damaging agents is provided (Hayman et al., 2021).

### 8.3 STING in metabolism

In the presence of *Brucella* infection, STING regulates the metabolic reprogramming of macrophages via HIF-1α involving oxidative phosphorylation and glycolysis (Gomes et al., 2021). It is then worth exploring whether the restoration of impaired respiratory function by tumor cells through horizontal transfer of mtDNA to enhance tumor-promoting behavior may also be related to STING, as mentioned above. The current study demonstrated the regulatory role of STING in glucose metabolism. Rong et al. found that in an environment of adequate nutrition and unactivated innate immunity, STING molecules could inhibit the autophagosome-lysosome fusion process involved in STX17 and downregulate autophagic flux by binding to STX17, an important protein for the fusion of autophagic membranes, and allowing it to reside in the ER. In the absence of STING, or upon activation by cGAMP, and ER-GIC translocation, STX17 is released from STING, leading to an upregulation of autophagy levels, which enhances AMPK activity in skeletal muscle cells (Rong et al., 2022). This reveals a novel non-immune function of STING, i.e., spatial modulation of the classical autophagy pathway induced by energy deprivation, suggesting a new link between innate immunity and energy metabolism. Tumor cells promote proliferation through aerobic glycolysis, or the Warburg effect, which is another hallmark of cancer. Tumor aerobic glycolysis produces an unfavorable TME, resulting in impaired anti-tumor immunity. Local nutrient depletion inhibits anti-tumor T-cells proliferation and activation, while accumulation of waste products (e.g., lactate) enhances the immunosuppressive TME (Wang et al., 2020b). Zhang et al. found that STING can target hexokinase II to block its activity, thereby promoting anti-tumor immunity by limiting aerobic glycolysis in tumor cells. In human colorectal cancer samples, lactate, which can be used as a proxy for aerobic glycolysis, was negatively correlated with STING expression levels and anti-tumor immunity (Zhang et al., 2023a). Collectively, these findings reveal a role for STING in the regulation of cellular metabolism and establish a critical link between the glycolytic regulation of STING and tumor suppressor activity.

### 8.4 STING in senescence

**FIGURE 2:**
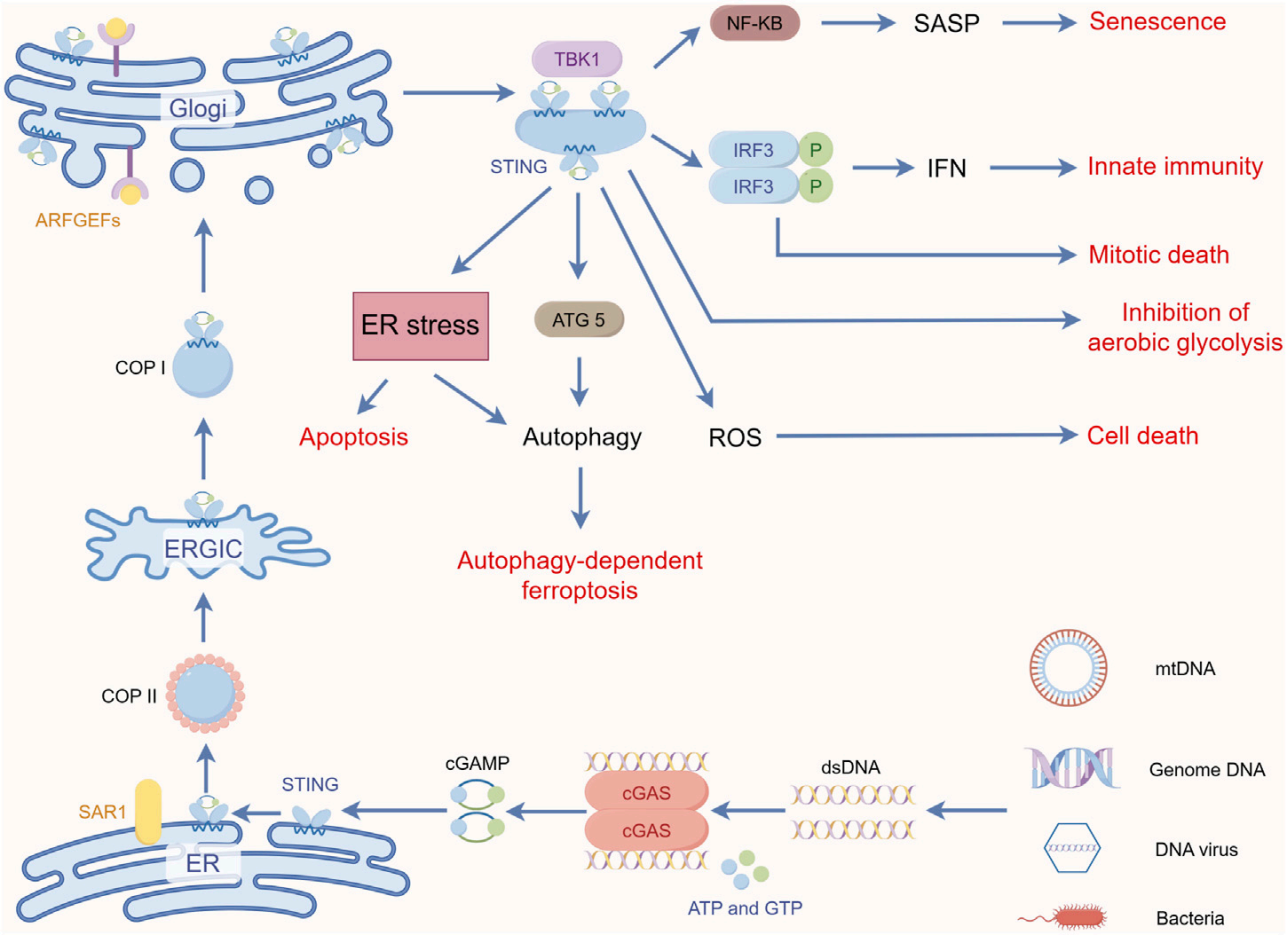
STING-mediated key non-innate immune pathways. By Figdraw.

STING, through activation of the downstream NF-κB pathway, also mediates the secretion of pro-inflammatory cytokines, chemokines, proteases, and growth factors, collectively referred to as SASPs (senescence-associated secretory phenotypes), which attenuate tumor growth by promoting cell cycle arrest in tumor cells (Dou et al., 2017; Yang et al., 2017). Yang et al. found that cGAS deletion accelerated spontaneous immortalization of mouse embryonic fibroblasts, and cGAS deletion also eliminated SASPs induced by multiple factors, including radiation and etoposide (Yang et al., 2017). These cytokines signaling programs can also induce enhanced inflammatory responses. For example, both IL-1β and IFN-α disrupt mitochondrial homeostasis to induce enhanced immune responses, demonstrating a novel function of IL-1β in initiating or enhancing STING-mediated intrinsic cellular immunity (Aarreberg et al., 2019). In this perspective, STING-mediated IFNs production and inflammatory factors generated by activation of the NF-κB pathway may further amplify the damaging effects of mitochondria, leading to increased mtDNA release and enhanced cGAS recognition, resulting in a cascade of amplified responses to inflammatory signals. Interestingly, it has recently been shown that NF-κB activation enhances STING signaling by modulating microtubule-mediated STING transport and that this synergistic interaction between NF-κB and STING triggers a cascade-amplified interferon response and robust host antiviral defense (Zhang et al., 2023b). In addition, Zhu et al. found that VDAC2 is a novel STING-binding ligand. STING deficiency or inhibition of the STING palmitoyltransferase ZDHHCs by 2-BP enhanced VDAC2/GRP75-mediated mitochondria-ER contact, increased mtROS/calcium levels, impaired mitochondrial function, and inhibited signaling in the mTOR pathway, which ultimately led to growth retardation in renal cell carcinoma (Zhu et al., 2023) (Figure 2). Table 1 summarizes the non-innate immunoregulatory pathways mediated by STING.

**TABLE 1:** Non-innate immune functions performed by cGAS-STING and their mechanisms.

Functionality	Mechanism	Pmcid
autophagy	Increased Drp1 promotes autophagy and ESCC progression by mtDNA stress mediated cGAS-STING pathway	35,209,954
	TFAM downregulation promotes autophagy and ESCC survival through mtDNA stress-mediated STING pathway	35,750,756
	GBP3-STING interaction in glioblastoma coordinates autophagy	37,204,260
Ferroptosis	Mitochondrial damage releases mtDNA, activates the cGAS-STING pathway, and induces autophagy-dependent ferroptosis	32,186,434
	STING increases mfn1/2-dependent mitochondrial fusion-induced ROS production causing ferroptosis	34,195,205
	Binding of cGAS localized on mitochondria to DRP1 promotes its oligomerization thereby inducing mitochondrial ROS accumulation and ferroptosis	36,864,172
Mitotic Cell Death	Mitotic inhibition activates the cGAS/STING pathway and induces apoptosis in breast cancer	31,937,780
	Slow accumulation of IRF3 phosphorylation by cGAS during mitotic arrest promotes cellular regulation through inhibition of Bcl-xL	31,299,200
DNA damage response	STING - TBK1 axis stimulates phosphorylation of kinase ATM, activates CHK2-p53-p21 pathway, and induces apoptosis due to cellular G1 phase blockade	34,707,113
glycolysis	STING targets hexokinase II and blocks its activity, limiting aerobic glycolysis in tumor cells to promote anti-tumor immunity	37,443,289
SASP	Cytoplasmic chromatin fragments accumulate in senescent cells and activate cGAS-STING-NF-κB signaling to promote SASP and cellular senescence	32,047,109
	nuclear porin Tpr activate the cGAS-STING pathway, and promoted the secretion of SASP in cancer cells	37,543,653
	cGAS-STING activation initiates oncogene-induced senescent TLR2 and A-SAAs expression to regulate SASP	31,183,403

## 9 The intensity of STING activation in tumor cells influences their outcome

Recent evidence suggests that cGAS-STING signaling may also be involved in the promotion of tumor proliferation and metastasis. Cheng et al. showed that in tumor cells, ROS induced by mitochondrial Lon, a chaperone and DNA-binding protein involved in protein quality control and stress response pathways, triggers mtDNA damage, and Ox-mtDNA is released into the cytoplasm to activate IFN signaling, which further upregulates the expression of programmed death ligand-1 (PD-L1) and indoleamine 2,3-dioxygenase (IDO-1) to inhibit T-cells activation. In addition, Lon upregulation induced the secretion of loaded mtDNA and PD-L1 EVs, which further induced the production of IFNs and IL-6 by macrophages, thereby attenuating innate and CD8^+^T cells immunity in the TME (Cheng et al., 2020). This process may be the mechanism for the formation of immunosuppressive TME in tumors, and mtDNA levels may serve as a potential diagnostic biomarker for anti-PD-L1 immunotherapy (Cheng et al., 2020). Zeng et al. also found that IL-6-induced ROS production in endometrial cancer (EC) cells and high levels of ROS cause cytoplasmic leakage of mtDNA and activate downstream cGAS-STING signaling, which increased PD-L1 levels. In IL-6-induced EC cell-derived EVs, mtDNA and PD-L1 expression levels were similarly significantly elevated (Zeng et al., 2022). Similarly, IR of radiotherapy activates innate immune pathways, leading to the upregulation of PD-L1 in hepatocellular carcinoma (HCC), inhibition of cytotoxic T lymphocytes activity and immune infiltration, and protection of tumor cells from cellular immune-mediated killing (Du et al., 2022).

**FIGURE 3:**
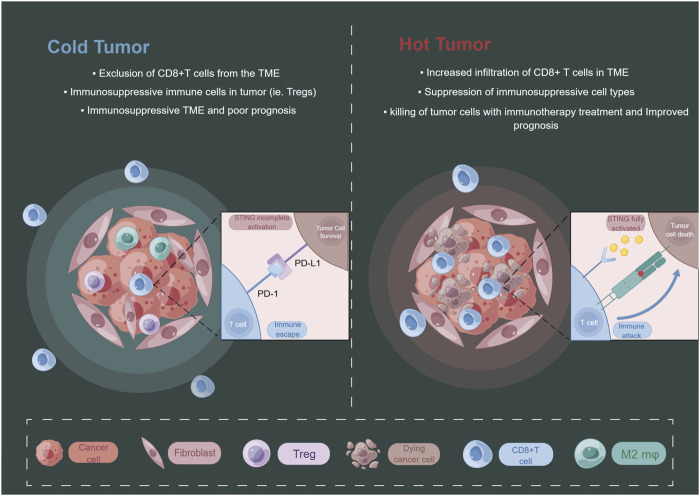
The level of STING activation in tumor cells may have an impact on the state of the tumor immune microenvironment. By Figdraw.

The emergence of this paradoxical phenomenon of STING may be related to its activation state, and its chronic activation may induce the formation of immunosuppressive TME (Coussens and Werb, 2002; Kwon and Bakhoum, 2020) (Figure 3). It has been shown that STING-deficient mice often develop more and larger tumors in advanced stages of HCC and show reduced levels of phosphorylated STAT1, autophagy, and cleaved caspase-3. Treatment with CDNs, a STING agonist, activates these responses in the liver, and CDNs treatment of advanced HCC in mice effectively reduces the size of most tumors (Thomsen et al., 2020). Application of engineered EVs exogenously loaded with CDNs preferentially activated APCs in the TME, enhanced local Th1 response and CD8^+^T cells infiltration in tumors as well as systemic anti-tumor immunity (Jang et al., 2021). Wang-Bishop et al. researchers mediated effective cytoplasmic delivery of the endogenous STING ligand cGAMP via intratumoral nanoparticle delivery, which enhanced STING activation relative to free cGAMP and improved response to PD-L1 immune checkpoint blockade, inducing immunogenic death in neuroblastoma (Wang-Bishop et al., 2020). Furthermore, the combination treatment of cGAMP with cisplatin exerted a stronger anti-tumor effect (Harabuchi et al., 2020).

Furthermore, the levels of intracellularly detectable DNA fragments are even more important for the degree of activation of the cGAS-STING pathway. Liu et al. recently found that STING signaling in CD11c^+^ dendritic cells is required for the anti-tumor effects of CD47 blockade therapy and that tumor cells clearance by CD47 blockade is dependent on the recognition of tumor cells cytoplasmic DNA. Impaired recognition of endogenous DNA, including mtDNA, would result in ineffective tumor cells clearance (Liu et al., 2015). Gusho et al. found significantly elevated cGAS levels in human papillomavirus (a dsDNA virus) positive cells that stably maintained viral-free bodies (Gusho and Laimins, 2022). Given the complex regulatory roles of STING and its downstream pathways in immunotherapy targeting tumor cells, the combination of STING agonists with immune checkpoint blockade therapies may be a better approach. This combination application could reduce the use of one drug and provide an economical, beneficial, and safe option for patients. For example, the combination of STING agonists and IDO inhibitors significantly inhibited tumor progression in a mouse colorectal cancer model compared to immune checkpoint therapy alone (Shi et al., 2021). Yi et al. combined the STING agonists and the anti-TGF-β/PD-L1 bispecific antibody successfully enhanced innate and acquired immunity in mice, promoted antigen presentation, improved T-cells migration and chemotaxis, and upregulated the number and activity of tumor-infiltrating lymphocytes, demonstrating new perspectives for overcoming immunotherapy resistance (Yi et al., 2022). Since mtDNA acts as a potent agonist of cGAS, facilitating the release of mtDNA into the cytoplasm may be another promising direction. However, this places greater demands on the understanding of the temporal and spatial mechanisms involved in the tumor-promoting or inhibitory effects of cGAS-STING, as well as on the ability to correctly localize this pathway. Since whether horizontally transferred mtDNA is “friend” or “foe” is determined by the role played by STING at the stage of tumor progression, if the release of mtDNA is not high enough to reach the threshold for full activation of STING, it is more likely that the tumor will choose the path of no return, driving malignant programs and inhibiting their anti-tumor functions, producing counterproductive effects. Therefore, it may be possible to choose to combine with STING agonists and immune checkpoint inhibitors for better therapeutic effects when perhaps necessary.

## 10 Conclusion

Under conditions of oxidative stress or impaired mitochondrial quality control, mtDNA escapes into intra- or extracellular compartments of the cytoplasm. Once shed, mtDNA acts as damage-associated molecular patterns (DAMPs) that bind danger-signaling receptors to trigger an innate immune response (Picca et al., 2020). The consequences of mtDNA also depend on the cellular context and cover a wide range of states, including triggering an innate immune response, inducing cell death, or acting as a tumorigenic agent. mtDNA follows MOMP into the cytoplasm. In the past, MOMP appeared to play a bipolar role in cells, i.e., as long as apoptotic signals are generated, they can be propagated to all mitochondria in cells, leading to apoptotic cell death. However, under sublethal stress conditions, a fraction of mitochondria undergoe MOMP without inducing cell death, the phenomenon known as minority MOMP (miMOMP) (Ichim et al., 2015). The escape of mtDNA from mitochondria under miMOMP conditions is worth exploring because tumor cells are constantly exposed to chronic stress conditions such as hypoxia, and thus mtDNA leakage induced by miMOMP under such sublethal stress conditions may be involved in the maintenance of cytoplasmic dsDNA in tumor cells as well as its delivery to the outside of the cells. This chronic stimulation of the cGAS-STING pathway of appropriate intensity in tumor cells may mediate tumor cells survival and metastasis. In addition, the transfer of mtDNA from tumor cells to surrounding immune cells via EVs or TnTs may trigger an apoptotic cascade in host immune cells, thus creating an “immunoparalyzing” microenvironment for tumor cells, which may partly explain the depletion of CD8^+^T cells in the immunosuppressive TME. There are still many unanswered questions about the pathway of mtDNA escape, including the lack of conclusive evidence for the increase in MIMP when mtDNA is extruded from MOMP. Further studies are needed on the influence of the GSDM family and mitochondrial dynamics aspects on mtDNA release.

The fact that cGAS-STING has complex consequences during tumor progression suggests that our understanding of the two sides of cGAS-STING in cancer progression is important for advancing cancer therapeutic strategies. As mentioned above, the use of STING agonists to induce tumor immunogenicity for anti-tumor therapy has achieved some efficacy. However, there is still a lack of detailed studies on individual differences in different tumors, stages, genotypes, *etc.*, as well as large sample data. These will determine the therapeutic response of tumor cells to STING agonists or antagonists. Admittedly, these studies have focused on the role of STING in innate immunity, and the exploration of the non-immune direction is currently very limited. Since STING modulation of processes such as tumor glycolysis, autophagy, and cell cycle has also shown anti-tumor effects, attempting to bypass the innate immunity aspect could prevent its potential for immune escape. For example, the atypical pathway autophagy process induced by STING participation is relatively independent and can be generated in the absence of TBK1 and IRF3 activation, and blocking IKK activation does not impair STING-induced autophagy (Gui et al., 2019). The cGAS-STING pathway can exert anti-tumor effects independent of IFNs and autophagy, which mainly depends on the recruitment ability of STING to TBK1. This suggests that the recruitment function of TBK1 to STING may be broader than the activation of IRF3 to promote IFNs synthesis (Yum et al., 2021). Overall, targeting STING will be a promising therapeutic prospect, whether focusing on the traditional innate immune aspects of STING or focusing on the emerging non-innate immune aspects of STING.
